# Ternary Cross-Modal Interactions between Sweetness, Aroma, and Viscosity in Different Beverage Matrices

**DOI:** 10.3390/foods9040395

**Published:** 2020-03-30

**Authors:** Anne Sjoerup Bertelsen, Line Ahm Mielby, Derek Victor Byrne, Ulla Kidmose

**Affiliations:** Department of Food Science, Faculty of Technical Sciences, Aarhus University, 8200 Aarhus N, Denmark; annesbertelsen@food.au.dk (A.S.B.); lineh.mielby@food.au.dk (L.A.M.); derekv.byrne@food.au.dk (D.V.B.)

**Keywords:** apple nectar, high-ester pectin, sensory interactions, sucrose, vanilla aroma

## Abstract

Sugar reduction in food and beverage products involves several challenges. Non-nutritive sweeteners may give unwanted off-flavors, while sugar-reduced products often lack mouthfeel. To overcome this, the addition of aroma to increase sweetness through cross-modal interactions, and the addition of hydrocolloids such as pectin to increase viscosity, have been suggested as strategies to aid sugar reduction. However, viscosity has been shown to decrease both taste and aroma intensities. An increase in viscosity may thereby affect the use of aromas as sweetness enhancers. Additionally, the effects of aromas and hydrocolloids on sweetness intensity and mouthfeel depend on the food matrix involved. The present study investigated cross-modal aroma–sweetness–viscosity interactions in two beverage matrices: water and apple nectar. The perceptual effects of vanilla aroma (0–1 mL/kg), sucrose (2.5%–7.5% *w*/*w*) and pectin (0%–0.3% *w*/*w*) were studied in both matrices. For each matrix, cross-modal interactions were analyzed with descriptive analysis using a trained sensory panel. The effect of vanilla aroma on sweetness intensity was found to be higher in apple nectar compared to in water. Furthermore, pectin affected neither taste, aroma, nor the cross-modal effects of aroma on taste in either of the matrices. These results indicate that pectin, in the studied range of concentrations, may be used to improve mouthfeel in sugar-reduced beverages, without compromising taste or aroma perception.

## 1. Introduction

Sugar reduction in food and beverages is of high relevance for individuals as well as for society, since high sugar consumption has been shown to contribute to obesity and related lifestyle diseases [1,2]. Moreover, the food and beverage industry is also very interested in the topic, due to the possible economic gains of designing successful sugar-reduced products. Non-nutritive sweeteners have been used extensively to replace sugar, but they often possess additional taste qualities considered as off-flavors. In addition, non-nutritive sweeteners also result in perceptually different temporal profiles compared to sucrose [3]. This may negatively affect consumers’ acceptability of the products [3,4]. Instead of using non-nutritive sweeteners, it has been suggested to use cross-modal interactions as an alternative strategy to reduce the sugar content in many food and beverages [5,6,7,8].

When stimuli in one sensory modality affect the perception of stimuli in another modality, it is called a cross-modal interaction [9], e.g., when the addition of aroma affects the perception of sweet taste [6,10,11,12,13,14,15,16,17]. The effect of aromas on taste perception is, among other things, dependent on the specific aromas used [16,18,19,20]. In particular, whether an added aroma is congruent with a given taste has been found to affect the aroma’s modifying effect on the perception of that taste. Congruency, which is believed to be a learned behavior [21], has been defined as “the extent to which two stimuli are appropriate for combination in a food product” [19]. Aromas that are associated with a given taste quality are most likely to enhance it. For instance, aromas described as smelling “sweet” and food-like have been found to enhance sweet taste intensity more than aromas described as smelling less “sweet” and less food-like [22].

Another perceptual problem with sugar reduced beverages is the lack of mouthfeel compared to sugar sweetened beverages [23]. Indeed, a reduction of added sugar in an orange nectar has been shown to significantly decrease perceived viscosity [24]. To attenuate this consequence, high-ester pectin has been used to increase the viscosity of, and improve mouthfeel in, beverages and soft drinks [25]. However, increased viscosity has previously been found to reduce the intensity of both taste [12,26,27,28,29] and aroma [30,31,32], even when aroma release was not affected [33,34]. Viscosity may therefore also be expected to affect cross-modal aroma–sweetness interactions, which could have consequences for the use of aromas as sweetness enhancers in sugar-reduced products. However, to our knowledge, only one study so far has evaluated ternary cross-modal interactions between sweetness, aroma and texture. Lethuaut et al. [34] investigated aroma–sweetness–texture interactions in model dairy desserts using three concentrations of sucrose (2.5%, 5.0% and 10.0% *w*/*w*), three concentrations of fruity aroma (4.5, 18 and 72 mg/kg) and four different textural agents (κ-carrageenan, ι-carrageenan, λ-carrageenan or an equi-mix of the three carrageenan types). The researchers found a significant interaction between sucrose and textural agent which affected sweet taste intensity. At 2.5% *w*/*w* sucrose, the different textural agents did not affect sweet taste intensity significantly differently, but at sucrose concentrations above 2.5% *w*/*w*, the dairy desserts prepared with λ-carrageenan were perceived significantly sweeter than desserts made with ι-carrageenan. Interestingly, they did not see an effect of aroma or interactions involving aroma influencing the intensity of sweet taste. Rather, they found that the textural agent as well as sucrose concentration influenced the perceived intensity of the aroma, even though the air–dessert partition coefficient did not change. However, as mentioned, this only occurred at the highest aroma concentrations tested [34]. Lethuaut and colleagues [34] thereby demonstrated that it is important to investigate all three modalities simultaneously.

The first objective of the current study was, therefore, to investigate aroma–sweetness–viscosity interactions by studying the effects of a high-ester pectin inducing low viscosity levels on aroma–sweetness interactions. Lethuaut et al. [34] commented that it could be due to the low level of congruency between the fruity aroma and the sweet taste in the dairy desserts investigated, that they did not observe any effects of aroma on sweet taste intensity. For the present study, we consequently chose an aroma that has previously been demonstrated to increase sweet taste multiple times in various matrices, namely vanilla aroma [6,7,16,17,35,36,37,38,39]. Even though vanilla has been found to increase sweet taste in different matrices, other studies have shown that the type of matrix may have big effects on sensory interactions [6,40]. Therefore, the second objective of this study was to investigate the effects of matrix on the aroma–sweetness–pectin interactions mentioned above. An experimental design was consequently set up to assess sensory interactions between vanilla aroma, sucrose and high-ester pectin in an aqueous model solution as well as in an apple nectar.

## 2. Materials and Methods

### 2.1. Design and Samples

A full factorial screening design was created in MODDE Pro (12.1.0.5491) (Sartorius Stedim Data Analytics AB, Umeå, Sweden) to test for perceptual interactions between vanilla aroma, sucrose and high-ester pectin. The design, which comprised 11 samples in total, is displayed in Table 1.

To investigate the effects of matrix, the design was tested in both local tap water as well as in a commercially available apple nectar, which was generously provided by Rynkeby Foods A/S, Ringe, Denmark. The ingredients in the apple nectar can be found in Table 2. Vanilla aroma was purchased from Bolsjehuset (Albertslund, Denmark) and sucrose from Merck KGaA (Darmstadt, DE). DuPont (Grindsted, Denmark) generously provided GRINDSTED® Pectin JD 470. This high-ester pectin was chosen because it showed the least flavor on its own, in comparison to other hydrocolloids in a pilot study. Pectin concentrations were also pilot tested to achieve a perceivable, but low increase of viscosity, at concentrations similar to commercial products [25]. Samples were produced in random order according to Table 1, by first adding either 700 g of water or apple nectar. If needed, pectin was then slowly added while stirring with a magnetic stirrer to dissolve the pectin. Sucrose, vanilla aroma (if needed), and the rest of the water or apple nectar was added afterwards. After production, samples were dispensed into opaque sample tubes with red lids (Fisher Scientific, Roskilde, DK) with 20 mL in each. Samples were stored for 1–3 days at 5 °C before sensory evaluation.

### 2.2. Sensory Evaluation

Descriptive analysis (DA) has previously been shown to identify aroma-related sensory interactions [41], and DA was therefore carried out for each matrix (water or apple nectar) with the experienced trained sensory panel in the sensory lab at the Department of Food Science, Aarhus University, Årslev, Denmark. The DA of the aqueous matrix was carried out first, followed by the DA of the apple nectar matrix two weeks later. For the aqueous matrix, the sensory panel consisted of two men and six women aged 41–60 years. For the apple nectar matrix, the panel consisted of two men and seven women, again aged 41–60 years. All panelists provided informed verbal consent prior to participation. Both DAs followed the same procedure, beginning with an introductory discussion where samples expected to span the relevant attributes were presented. Based on the initial discussion, the panel chose the following attributes for the aqueous matrix (evaluated in the order shown): sweet aroma, vanilla aroma, synthetic fruit aroma, sweet taste, vanilla flavor and stale off-flavor. The attributes chosen for the apple nectar matrix were: apple juice aroma, vanilla aroma, sweet aroma, acidic aroma, sweet taste, acidic taste, apple flavor and vanilla flavor. For both the aqueous matrix and the apple nectar matrix, aroma attributes were evaluated orthonasally and flavor attributes were evaluated retronasally. In neither of the two matrices did the panel choose to include viscosity as an attribute in the DA. The perception of viscosity was therefore not measured. The discussion was followed by training on a selected subset of samples, and then, the final evaluation of the entire sample set. During both the training and the final evaluation, samples were assessed in triplicate and served at room temperature. The final evaluation took place over two consecutive days. Samples were coded with three-digit numbers and presented to panelists according to a Williams Latin Square design in order to limit carry over effects [42]. Data were collected using Compusense (Compusense Inc., Ontario, Canada). For each attribute, panelists evaluated the samples on a 15 cm line scale with two anchor points indicating low (= 1.5) and high (= 13.5) intensity, respectively. Between the samples, panelists were instructed on the screen to cleanse their mouth and to wait at least 30 s before beginning the evaluation of the next sample. Water; tepid, very weak white tea and crackers were used for mouth cleansing.

### 2.3. Viscosity Measurements

After the sensory evaluations, the viscosity of each sample was measured using a Brookfield DVELV viscosimeter with a UL adapter (Buch & Holm A/S, Herlev, Denmark). Measurements were performed at 60 rpm at 19 ± 1 °C, in random order and in duplicate.

### 2.4. Statistical Analyses

To determine the effect of each factor (sucrose, aroma and pectin) on the sensory attributes as well as instrumentally measured viscosity, the design was analyzed for each matrix (water and apple nectar) with multiple linear regression in MODDE Pro (12.1.0.5491) (Sartorius Stedium Data Analytics AB, Umeå, Sweden) using the averages for each sample. All models were stepwise reduced to include only significant terms (*p* ≤ 0.05). Principal Component Analysis (PCA) of the mean centered average results from the DAs were also performed, and the average results from the viscosity measurements were added to the PCA biplots as supplementary variables (XLSTAT (2018.5) (Addinsoft, NY, USA) [43]). Finally, a three-way analysis of variance (ANOVA) and Tukey’s HSD multiple comparison test were used to find significant differences between samples at 5% significance level for sweet taste intensity in each matrix (carried out in XLSTAT). In the ANOVAs, samples were considered to be fixed effects, while panelists and replicates were considered to be random effects.

## 3. Results

We studied the interactions between sucrose, aroma and pectin in an aqueous matrix and in an apple nectar matrix, respectively. First, the results from the aqueous matrix are presented, followed by the results from the apple nectar matrix. Finally, the results for sweet taste intensity in both matrices are compared in more detail.

### 3.1. Effects of Design Factors in the Aqueous Matrix

**Figure 1:**
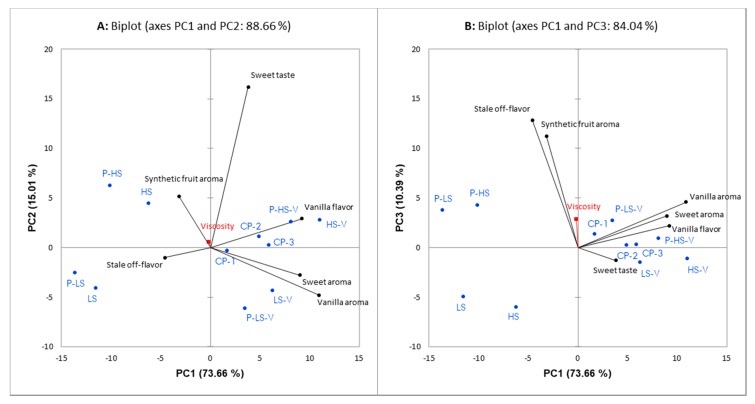
Principal Component Analysis (PCA) biplots of the results from the Descriptive Analysis (DA) of the aqueous matrix. PC1 versus PC2 (**A**) and PC1 versus PC3 (**B**) is shown. PC1 explains 73.66%, PC2 15.01%, and PC3 10.39% of the variation in the data. Attributes are marked in black, samples in blue, and the supplementary variable viscosity in red. Samples are labelled according to Table 1.

To get an overview of how the attributes in the DA were related, a PCA was carried out. In Figure 1, PCA biplots are shown for the aqueous matrix. Principle Component (PC) 1 explains 73.66%, while PC2 explains 15.01% and PC3 explains 10.39% of the variation in the data from the DA. As seen in Figure 1, the intensity of sweet taste is mainly explained by PC2, while the intensities of the attributes sweet aroma, vanilla aroma and vanilla flavor are positively correlated and mostly explained by PC1. The synthetic fruit aroma and stale off-flavor are positively correlated and mostly explained by PC3.

From the linear regression, it was determined which design factors that significantly affected the attributes in the DA, as well as the viscosity measurements. Table 3 displays the coefficients for the significant design factors for the aqueous matrix. It shows that vanilla aroma concentration significantly affected all attributes in the DA, including the evaluation of sweetness, thus demonstrating a cross-modal effect. As expected, the effect of sucrose concentration on sweet taste intensity was higher than the effect of vanilla aroma concentration on sweet taste intensity (Figure 1, Table 3).

Pectin (*p* < 0.0001) and sucrose (*p* = 0.04) concentrations significantly affected viscosity measurements (Table 3). However, in both biplots (Figure 1), viscosity is loaded close to the origin, which means it does not explain much of the variance between the samples from a sensory perspective. In addition to viscosity, the pectin concentration significantly affected the panel’s evaluation of synthetic fruit aroma (*p* < 0.0001) and stale off-flavor (*p* < 0.001) (Figure 1, Table 3). Only one significant interaction (*p* < 0.001) was found between the design factors, namely between aroma and pectin, which affected the intensity of synthetic fruit aroma (Table 3). For the samples without pectin, the intensity of synthetic fruit aroma was evaluated similarly, independent of whether or not aroma was present (1.4). However, at high pectin concentrations, the intensity of the synthetic fruit aroma was evaluated higher for the samples without aroma (8.7) than for the samples with addition of the high aroma concentration (2.3). As the three center points (CP) were made of the medium concentrations of all three design factors, they could be expected to give average intensities for the different sensory attributes and are accordingly found close to the origin of both biplots (Figure 1).

### 3.2. Effects of Design Factors in the Apple Nectar Matrix

**Figure 2:**
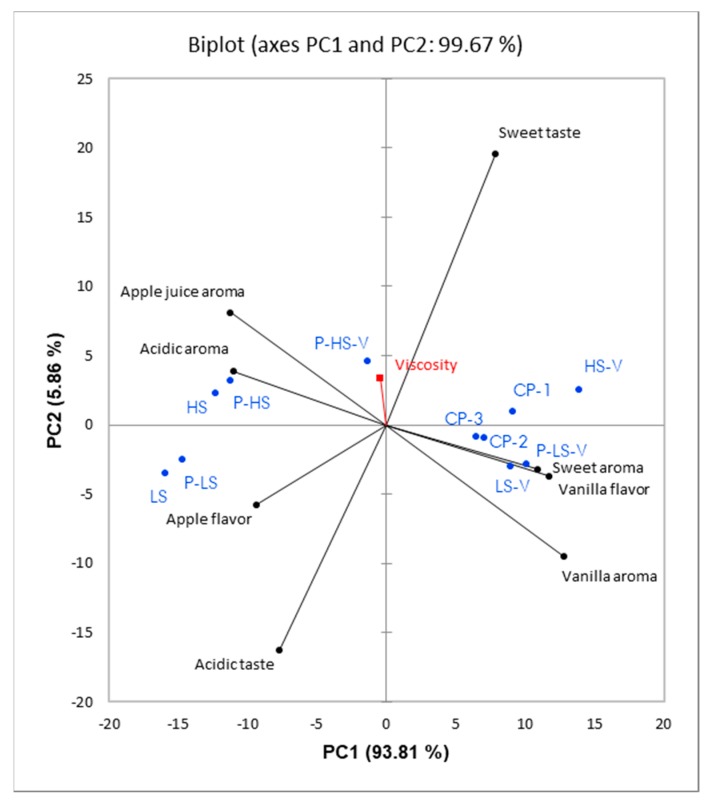
Principal Component Analysis (PCA) biplot of the results from the Descriptive Analysis (DA) of the apple nectar matrix. PC1 explains 93.81% of the variation and PC2 explains 5.86% of the variation in the data. Attributes are marked in black, samples are marked in blue, and the supplementary variable viscosity is marked in red. Samples are labelled according to Table 1.

In Figure 2, a PCA biplot from the results for the apple nectar matrix is shown. PC1 and PC2 explain 93.81% and 5.86% of the variation in the data, respectively. As for the aqueous matrix, the sweet taste intensity is mainly explained by PC2, while the intensities of sweet aroma, vanilla aroma, and vanilla flavor are positively correlated and mostly explained by PC1. In contrast to the aqueous matrix, these aroma attributes are negatively correlated with the intensity of apple juice aroma and acidic aroma, whereas the sweet taste intensity is negatively correlated to acidic taste intensity. Thereby, the data show both a sweet-acidic aroma and taste dimension.

The coefficients for the significant factors affecting the attributes from the DA as well as the viscosity in the apple nectar matrix are shown in Table 4. In accordance with the aqueous matrix, vanilla aroma concentration significantly affected all attributes in the DA including sweet taste intensity. A cross-modal effect on sweet taste intensity is thereby again present.

Contrary to the aqueous matrix — as seen from the sucrose and aroma coefficients for sweet taste — the effect of vanilla aroma concentration on sweet taste intensity was in the apple nectar matrix higher than the effect of sucrose concentration on sweet taste intensity. Similar to the aqueous matrix, pectin (*p* < 0.0001) and sucrose (*p* = 0.04) concentrations significantly affected viscosity in the apple nectar matrix (Table 4). Viscosity is, however, again loaded close to the origin of the biplot and therefore does not explain the intensity differences between the samples in the DA (Figure 2). Contrary to the aqueous matrix, where pectin concentration also affected the intensity of two attributes in the DA, pectin concentration only significantly influenced viscosity in the apple nectar matrix. Furthermore, no significant interactions between the design factors were identified in the apple nectar matrix (Table 4).

### 3.3. Comparison of Sweet Taste Intensity Between Matrices

**Figure 3:**
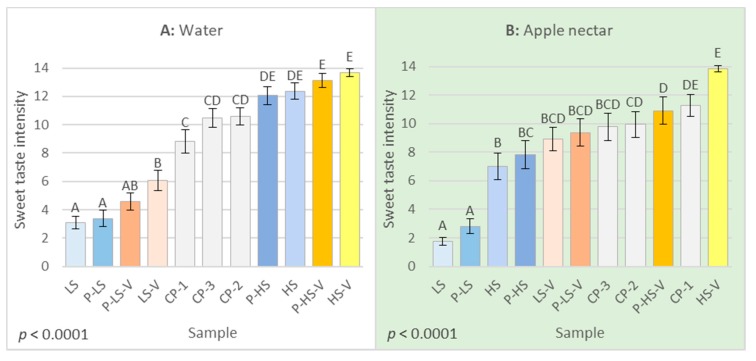
Means of sweet taste intensity ± std. error of the mean for each sample in the Descriptive Analysis (DA) of the aqueous matrix (**A**) and the apple nectar matrix (**B**). Different letters indicate significant (*p* ≤ 0.05) differences between samples in each matrix. Samples are labelled according to Table 1.

Figure 3 compares the means of sweet taste intensities for each sample in the aqueous and apple nectar matrix, respectively. For both matrices, the three replicates consisting of the center points were not significantly different from each other, thereby demonstrating repeatability in both matrices. In the aqueous matrix, the four samples with the highest concentration of added sucrose were evaluated as most sweet, though not significantly different from each other. Similarly, the samples with the lowest concentration of sucrose added were evaluated as the least sweet. The sample LS-V — with the addition of low concentration sucrose, high concentration vanilla aroma and no pectin — was, however, significantly sweeter than samples LS and P-LS, both with low concentration sucrose and no vanilla aroma. This demonstrates a cross-modal effect of vanilla aroma on sweet taste intensity at the low sucrose concentration, but not at the high sucrose concentration.

In the apple nectar matrix, sample HS-V, with added high sucrose and vanilla aroma concentrations and no pectin, was again evaluated the sweetest, though in this matrix, it was significantly sweeter than sample P-HS-V, added high concentrations of all three design factors. The addition of pectin here seemed to reduce sweet taste intensity as the only time in both matrices. In the apple nectar, sample P-HS-V was placed in between the three center points regarding sweetness intensity. Samples LS-V and P-LS-V, both with added low sucrose concentration and high vanilla aroma concentration, were significantly different from the three center points in the aqueous matrix but not different in the apple nectar matrix. Most drastically, samples HS and P-HS, both with added high sucrose concentration and no aroma, had shifted from being evaluated very sweet to not very sweet. Samples HS and P-HS were in the apple nectar matrix only evaluated sweeter than samples LS and P-LS, with low sucrose concentration and no aroma added. A cross-modal effect of vanilla aroma on sweet taste intensity was thereby seen both at the low and high sucrose concentrations in the apple matrix, compared to only the low sucrose concentration in the aqueous matrix.

## 4. Discussion

The cross-modal interactions between sucrose, vanilla aroma and pectin were investigated in an aqueous matrix as well as in an apple nectar matrix. First, the results for the cross-modal effect of vanilla aroma on sweet taste intensity will be discussed followed by a discussion of the effects of high-ester pectin on aroma and taste intensities. Finally, the differences between the two matrices will be presented.

### 4.1. The Cross-Modal Effect of Vanilla Aroma on Sweet Taste Intensity

In both matrices, a significant cross-modal effect of the vanilla aroma was seen on sweet taste intensity. The vanilla aroma was in both matrices found to enhance sweet taste with increasing vanilla aroma concentrations (Table 3 and Table 4), at least up to 1 mL/kg which was the maximum concentration tested in this study. As mentioned, this is in accordance with an abundance of previous literature demonstrating that addition of vanilla aroma increased sweet taste perception [6,7,16,17,35,36,37,38,39]. However, in terms of vanilla concentration, few authors have studied the effect of vanilla aroma concentration on sweet taste intensity. Labbe et al. [6] found that only 0.1% and not 0.05% vanilla flavoring increased sweet taste intensity in a familiar cocoa beverage, while Wang et al. [17] for both 1.31% and 2.24% *w*/*w* sucrose in milk found stronger interactions between sucrose and vanilla affecting sweet taste intensity at lower versus higher vanilla concentrations. Hence, our study is mostly in accordance with the study by Labbe et al. [6]. The discrepancy in the results may be due to the different types of vanilla aromas used in the different studies. Indeed, Velázquez et al. [44] recently found that only the addition of one of two vanilla aromas tested significantly increased sweetness intensity of vanilla milk desserts. As in the present study, Labbe et al. [6] used a commercially available vanilla aroma, while Wang et al. [17] used a vanilla extract.

Other authors have investigated the effect of aroma concentration on sweetness enhancement for different aromas. Similarly to Wang et al. [17], Stevenson et al. [22] found that for lychee aroma, only the lowest concentration (out of 5) was found to increase the sweet taste intensity of a 0.3 M sucrose solution. In accordance with Labbe et al. [6] and the present study, others have found that increasing concentrations of congruent aromas have also increased sweet taste enhancement [19,45]. The lychee aroma tested by Stevenson et al. [22] was relatively unfamiliar, which may explain the differences seen when compared to other studies. Results are, however, generally difficult to compare as the studies have tested various concentrations, aromas, matrices and methods.

In terms of the effect of vanilla aroma in relation to the concentration of sucrose, the addition of vanilla aroma only significantly increased the sweet taste intensity at the low sucrose concentration in the aqueous matrix (Figure 3A). The fact that the cross-modal effect of aroma on sweet taste intensity is more pronounced at lower sucrose concentrations is also in accordance with previous literature [17,19,20,39,46]. However, this is not in accordance with the results from the apple nectar matrix, where the addition of vanilla aroma increased sweet taste intensity independently of the sucrose concentration (Figure 3B). Furthermore, the base product of the apple nectar matrix contained 9.8% *w*/*v* sugar, which is why a cross-modal effect at the high sucrose concentration is even more surprising. The apple nectar, however, also contained organic acids contributing with sour taste. Since sour taste may suppress sweet taste through taste-taste interactions [47] this may help explain why the addition of vanilla aroma still enhanced sweet taste intensity at the high sucrose concentration in the apple nectar matrix.

### 4.2. The Effects of High-Ester Pectin on Aroma and Taste Intensities

According to the DAs of the two matrices, there was, interestingly, only an effect of pectin on the intensity of the synthetic fruit aroma and stale off-flavor in the aqueous matrix (Table 3). Even though the pectin was chosen due to its low intrinsic flavor, it clearly still had both an aroma and flavor by itself. However, these were seemingly masked and thereby not detected by the panel in the apple nectar matrix (Table 4). The addition of pectin was only found to affect sweet taste intensity significantly at the high sucrose concentration in the apple nectar matrix (Figure 3B), where it reduced the sweet taste intensity. The sample P-HS-V, which was the sample with the highest concentration of all design factors, was however evaluated similar to the three center points in terms of sweetness intensity (Figure 3B) and close to the origin of the PCA biplot (Figure 2). The sample P-HS-V therefore seems to have behaved differently than the rest of the samples in the apple nectar matrix. The suppressing effect of pectin on sweet taste intensity is, in this instance, hence questionable. The fact that pectin only affected the intensities of the synthetic fruit aroma and stale off-flavor in the aqueous matrix is in contrast to other findings. Both taste [12,26,27,28,29] and aroma [30,31,32,33,34], have been found to decrease when viscosity increased. That only few effects of pectin were seen, even though pectin was still found to increase viscosity, is probably due to the relatively low concentration of pectin used.

The study by Kappe et al. [48] suggested that even viscosity differences as low as 0.527 cp may be detected by consumers in beverages. Even though the pectin concentrations in the present study were pilot tested to be perceivable, and the viscosity increase from adding the high concentration pectin (but not sucrose) was higher than 0.527 cp, the absolute viscosities were generally below 16 cp. A viscosity of 16 cp is necessary to reduce sweetness intensity of sucrose significantly [28]. Comparing fruit drinks with 20, 40 and 70 cp, Brandenstein et al. [49] found, however, that for most samples, viscosity did not significantly affect sweetness intensity. Although the viscosities in the present study were lower than viscosities normally found to reduce sweet taste intensity, viscosity could possibly have affected sweet taste intensity, or the cross-modal effect of vanilla aroma on sweet taste intensity, due to a central integration of inputs from different senses. Indeed, taste have been shown to increase sensitivity of congruent aromas even at sub-threshold concentrations [50,51].

### 4.3. The Effects of Matrix on Sweetness-Aroma-Pectin Interactions

A cross-modal effect of vanilla aroma on sweet taste intensity was seen both at the high and low sucrose concentration in the apple nectar matrix (Figure 3B), compared to only the low sucrose concentration in the aqueous model (Figure 3A). This is in contrast to previous studies showing that sensory interactions are more discernible in aqueous models than more complex models [40,52]. Looking at aroma-induced sweetness enhancement, Barba et al. [52], for example, reported that out of nine aromas associated with sweetness, two aromas significantly increased sweetness of 7% sucrose in water, while six others showed a trend to increase the sweetness. However, in a fruit juice with 7% sucrose, only one of the aroma compounds significantly increased sweetness [52]. The fact that we still saw more aroma-induced sweetness enhancement in the apple nectar matrix compared to the aqueous matrix may be due to familiarity. Labbe et al. [6] investigated the effect of food matrix with respect to familiarity on aroma–taste interactions. They reported that vanilla aroma increased sweetness intensity in a familiar, bitter, cocoa beverage, while it did not in an unfamiliar, bitter, caffeinated milk beverage. The results in the present study may therefore be due to the aqueous model being more unfamiliar to the panelists than the apple nectar matrix.

In water, the effect of sucrose concentration was higher than the effect of vanilla aroma concentration on sweet taste intensity (Table 3), while it was the opposite for the apple nectar (Table 4). Results from the aqueous matrix are in accordance with previous literature suggesting that sweetness is increased more by sweeteners than aromas [8,17,45]. Owing to the sugars already present in the base product of the apple nectar matrix, the lower effect of sucrose on sweet taste intensity in this matrix may have been due to a higher baseline sweetness of the matrix in general. Thus, the relatively low effect of sucrose on sweet taste intensity in the apple nectar matrix could be because the dose-response curve was flattening at higher concentrations. Indeed, recent studies have shown that sucrose as well as most alternative sweeteners, both nutritive and non-nutritive ones, follow sigmoidal dose-response functions [53,54]. However, in both cases, the essentially linear part of the curve for sucrose was roughly around 6%–18% *w*/*v* [53,54]. With 9.8% *w*/*v* sugar present in the base product and 2.5%–7.5% *w*/*w* sucrose added, the total amount of sugar in the samples is not likely to have exceeded the amount of 18% *w*/*v* sugar, thereby entering the non-linear flattening part of the curve. In addition, the acids from the base product are expected to suppress the sweet taste intensity [47], which may have attenuated the higher level of sweetness in the apple nectar matrix. Generally, differences between matrices could be due to various possible interactions between the factors tested and the components already present in the apple nectar (such as apple aroma or acids) [55]. The viscosity was also generally higher in the apple nectar matrix as compared to aqueous matrix, which may have affected the release of aroma compounds [56,57,58], although the viscosity from the pectin was not found to affect the intensity of any of the sensory attributes in the apple nectar matrix.

## 5. Conclusions

In order to aid sugar reduction in beverages, the present study investigated cross-modal interactions between sucrose, vanilla aroma and pectin in an aqueous matrix, as well as in an apple nectar matrix. The effect of vanilla aroma on sweet taste intensity increased with concentration, and interestingly, the relative effect was seen to be higher in the apple nectar matrix than in the aqueous matrix, possibly due to the apple nectar matrix being more familiar than the aqueous matrix. The effect of adding low concentrations of high-ester pectin to improve mouthfeel did not seem to affect possible cross-modal aroma–sweetness interactions involving vanilla aroma, in either water or apple nectar. Although the effect of viscosity on aroma–sweetness interactions were investigated in a non-sugar reduced beverage functioning as a model system, the results suggest that using high-ester pectin to improve mouthfeel does not interfere with the use of aroma to aid in sugar reduction in beverages.

## Figures and Tables

**Table 1:** Experimental screening design tested in both matrices, water and apple nectar, including pectin, sucrose and vanilla concentration.

Sample	Pectin (% *w*/*w*)	Sucrose (% *w*/*w*)	Vanilla Aroma (mL/kg)
LS	0	2.5	0
P-LS	0.3	2.5	0
HS	0	7.5	0
P-HS	0.3	7.5	0
LS-V	0	2.5	1
P-LS-V	0.3	2.5	1
HS-V	0	7.5	1
P-HS-V	0.3	7.5	1
CP-1	0.15	5.0	0.5
CP-2	0.15	5.0	0.5
CP-3	0.15	5.0	0.5

LS = low sucrose concentration, HS = high sucrose concentration, P = pectin, V = vanilla aroma, CP = center point.

**Table 2:** Lists of content of the commercial apple nectar used as a base product.

Content of the Commercial Apple Nectar (Rynkeby Foods A/S)	Nutritional Content of the Commercial Apple Nectar (per 100 mL)
Apple juice (55%) from concentrate, water, lemon juice from concentrate and sugar.	Energy 40 kcal, Fat < 0.5 g, Carbohydrates 10 g (of which 9.8 g is sugars), protein < 0.5 g and salt 0.02 g.

**Table 3:** Scaled and centered coefficients for significant main effects and 2-way interactions as well as means ± std. error of the mean for the samples in the aqueous matrix. Viscosity means are in cP. Samples are labelled according to Table 1.

Water	Descriptive Analysis						
	Sweet Aroma	Vanilla Aroma	Synthetic Fruit Aroma	Sweet Taste	Vanilla Flavor	Stale Off-Flavor	Viscosity
**Sucrose**	-	-	0.54	4.26	1.75	-	0.13
**Aroma**	4.63	5.72	−1.60	0.82	4.30	−1.79	-
**Pectin**	-	-	2.05	-	-	2.80	0.72
Sucrose*aroma	-	-	-	-	-	-	-
Sucrose*pectin	-	-	-	-	-	-	-
Aroma*pectin	-	-	−1.63	-	-	-	-
**LS**	1.06 ± 0.23	0.60 ± 0.11	1.00 ± 0.22	3.08 ± 0.44	0.91 ± 0.14	6.14 ± 1.17	1.17 ± 0.01
**P-LS**	1.78 ± 0.36	0.96 ± 0.21	7.50 ± 1.08	3.39 ± 0.57	0.97 ± 0.12	12.52 ± 0.48	2.47 ± 0.06
**HS**	3.04 ± 0.79	1.53 ± 0.41	1.83 ± 0.61	12.37 ± 0.56	3.52 ± 0.88	3.60 ± 0.96	1.31 ± 0.00
**P-HS**	2.33 ± 0.60	1.27 ± 0.36	10.01 ± 0.97	12.05 ± 0.64	3.67 ± 0.96	11.20 ± 0.93	2.74 ± 0.02
**LS-V**	11.18 ± 0.85	12.18 ± 0.64	1.23 ± 0.25	6.08 ± 0.71	9.74 ± 0.88	2.90 ± 0.79	1.14 ± 0.01
**P-LS-V**	10.93 ± 0.65	12.53 ± 0.55	2.06 ± 0.52	4.58 ± 0.59	7.65 ± 0.76	8.33 ± 1.04	2.48 ± 0.01
**HS-V**	12.30 ± 0.50	13.16 ± 0.50	1.71 ± 0.55	13.67 ± 0.29	13.65 ± 0.29	2.47 ± 0.82	1.30 ± 0.01
**P-HS-V**	10.85 ± 0.78	12.28 ± 0.58	2.56 ± 0.77	13.11 ± 0.50	12.41 ± 0.55	5.45 ± 1.08	2.96 ± 0.04
**CP-1**	8.51 ± 0.94	9.14 ± 0.99	3.91 ± 0.83	8.82 ± 0.80	8.67 ± 0.81	6.86 ± 1.18	1.66 ± 0.03
**CP-2**	9.71 ± 0.94	9.98 ± 0.98	3.23 ± 0.91	10.56 ± 0.60	10.97 ± 0.64	5.02 ± 0.93	1.74 ± 0.01
**CP-3**	10.28 ± 0.86	11.68 ± 0.80	2.73 ± 0.75	10.47 ± 0.68	10.18 ± 0.83	4.96 ± 0.95	1.71 ± 0.02

**Table 4:** Scaled and centered coefficients for significant main effects and 2-way interactions as well as means ± std. error of the mean for the samples in the apple nectar matrix. Viscosity means are in cP. Samples are labelled according to Table 1.

Apple Nectar	Descriptive Analysis								
	Apple Juice Aroma	Vanilla Aroma	Sweet Aroma	Acidic Aroma	Sweet Taste	Acidic Taste	Apple Flavor	Vanilla Flavor	Viscosity
**Sucrose**	-	-	-	-	2.09	−1.70	-	-	0.16
**Aroma**	−4.01	4.48	4.04	−3.91	2.96	−2.92	−3.49	4.21	-
**Pectin**	-	-	-	-	-	-	-	-	1.16
Sucrose*aroma	-	-	-	-	-	-	-	-	-
Sucrose*pectin	-	-	-	-	-	-	-	-	-
Aroma*pectin	-	-	-	-	-	-	-	-	-
**LS**	12.84 ± 0.49	0.85 ± 0.10	1.75 ± 0.22	12.57 ± 0.54	1.75 ± 0.29	13.30 ± 0.34	13.17 ± 0.51	1.16 ± 0.34	1.56 ± 0.02
**P-LS**	12.60 ± 0.49	1.23 ± 0.27	1.95 ± 0.23	12.35 ± 0.38	2.81 ± 0.53	12.66 ± 0.38	12.15 ± 0.55	1.43 ± 0.41	3.74 ± 0.01
**HS**	12.91 ± 0.37	0.91 ± 0.12	2.74 ± 0.59	11.99 ± 0.62	6.99 ± 0.93	9.34 ± 0.87	11.41 ± 0.78	1.41 ± 0.47	1.74 ± 0.00
**P-HS**	12.64 ± 0.40	0.99 ± 0.14	2.61 ± 0.58	11.86 ± 0.61	7.82 ± 0.97	8.55 ± 0.79	10.60 ± 0.77	2.10 ± 0.57	4.10 ± 0.02
**LS-V**	3.51 ± 0.82	11.51 ± 0.91	11.29 ± 0.85	3.43 ± 0.87	8.91 ± 0.83	6.87 ± 0.90	5.07 ± 0.72	10.50 ± 0.87	1.51 ± 0.00
**P-LS-V**	2.92 ± 0.75	11.98 ± 0.85	11.40 ± 0.85	3.20 ± 0.82	9.38 ± 0.96	6.14 ± 0.89	5.20 ± 0.82	11.32 ± 0.92	3.79 ± 0.00
**HS-V**	2.80 ± 0.56	12.02 ± 0.74	11.91 ± 0.67	2.90 ± 0.68	13.84 ± 0.24	2.25 ± 0.43	2.49 ± 0.51	12.32 ± 0.72	1.79 ± 0.01
**P-HS-V**	9.70 ± 0.93	4.34 ± 0.91	6.73 ± 0.94	7.94 ± 1.02	10.91 ± 0.96	5.23 ± 0.93	6.69 ± 0.92	5.64 ± 1.03	4.25 ± 0.01
**CP-1**	4.85 ± 0.84	10.31 ± 0.95	9.98 ± 0.91	3.29 ± 0.70	11.27 ± 0.77	4.39 ± 0.74	3.96 ± 0.63	10.43 ± 0.86	2.54 ± 0.01
**CP-2**	4.84 ± 0.94	10.16 ± 0.92	9.83 ± 0.90	3.86 ± 0.82	9.94 ± 0.90	6.09 ± 0.93	5.70 ± 0.83	9.56 ± 0.90	2.44 ± 0.02
**CP-3**	4.94 ± 0.98	9.90 ± 1.09	9.94 ± 1.00	4.50 ± 0.89	9.79 ± 0.95	6.17 ± 0.83	5.74 ± 0.86	9.19 ± 1.08	2.50 ± 0.01
